# Accuracy of digital indirect bonding technology for customized orthodontic brackets based on personalized typodonts

**DOI:** 10.1186/s12903-025-05777-x

**Published:** 2025-04-03

**Authors:** Yuqian Li, Linyi Zhou, Menghan Chen, Yiyang Du, Yuyang Gan, Biyao Li, Jianying Feng

**Affiliations:** School/Hospital of Stomatology, Zhejiang Chinese Medical University, No. 548, Binwen Rd, Hangzhou, Zhejiang 310053 China

**Keywords:** Digital indirect bonding technology, Customized orthodontic system, Personalized typodont, Accuracy

## Abstract

**Objective:**

The chief aim of this study was to confirm the accuracy and repeatability of digital indirect bonding (IDB) by simulating customized clinical orthodontic procedures with personalized typodonts from the perspective of orthodontic outcomes.

**Methods:**

Five personalized typodonts were produced with 3D-printing technology to mimic straight-wire orthodontic procedures. The digital IDB was employed to position the customized brackets. After treatment, the PAR index, the ABO-OGS index and the occlusal contact area were analyzed. The matching degree between the target position and the posttreatment position on typodonts was assessed with Geomagic Control X.

**Results:**

The mean arch discrepancy between the personalized typodonts and the initial intraoral scan model was 0.15 mm ± 0.01 mm in the maxilla and 0.20 mm ± 0.01 mm in the mandible. Following customized orthodontic therapy, the PAR Index decreased from 29 to 1, the ABO-OGS Index was 13.8 ± 0.84, and the occlusal contact area increased 4.04mm^2^ ± 1.14mm^2^, with the bilateral occlusal contact area becoming equally distributed. The mean arch discrepancy between the target position and the actual posttreatment positions was 0.15 mm ± 0.01 mm in the maxilla and 0.16 mm ± 0.01 mm in the mandible.

**Conclusions:**

Digital IDB is conducive to locating the brackets in the target position to precisely achieve the ideal therapeutic outcome of customized orthodontic systems on the personalized typodont. The customized bracket design and the digital IDB can lead orthodontics in a more accurate, visual, and predictable direction.

## Introduction

Over the past several decades, advancements in digital technology have revolutionized orthodontic diagnosis and treatment, making them more precise, personalized, and predictable [1]. Customized orthodontic brackets and digital IDB emerged. Customized orthodontic systems determine target tooth positions through virtual tooth movement in three dimensions based on individual dental structures, arch characteristics, and facial aesthetics. Depending on the determined target positions, the axial inclination, torque, and baseplate thickness of the brackets and the individual archwires are specifically designed and then 3D-printed [2]. This technique offers numerous benefits, such as highly predictable outcomes, precise bracket data expression, and fewer orthodontic risks [3]. As a crucial part of customized orthodontic systems, the digital IDB fabricates corresponding trays through 3D-printing based on the virtual positions of brackets and the morphology of crowns [4]. This technology not only enhances the precision of bracket positioning but also reduces chairside operating time and improves patient comfort [5, 6]. As digital dentistry and 3D-printing technology continue to advance, alongside the accumulation of supporting clinical evidence, customized orthodontic systems may become increasingly popular [7].

Wang et al. [8] found that following orthodontic treatment with the fully customized labial appliances (FORMULA; Shanghai Jing-gong Dental Technology Co., Ltd., Shanghai, China), the mean realization ratio between the expected and the actual dental arches was as high as 96-97%. A similar conclusion was drawn by Muller-Hartwich et al. [9] with customized archwires using the Sure-Smile system (OraMetrix; Richardson, TX, USA). More clinical data are required to further verify the matching precision between the target positions and the clinical outcomes of customized orthodontic treatments. Extensive accuracy studies of digital IDB were focused on three-dimensional localization analysis of the intraoral transferred positions and the intended positions of brackets [10–12]. However, clinical and laboratory evaluations for precision analysis according to therapeutic goals and outcomes have been rarely studied. The finite element method (FEM) [13, 14] is not fully capable of replicating the actual clinical situation, which is influenced by factors such as the aeolotropism and nonlinear elasticity of multiple stress variables, including teeth, alveolar bone, orthodontic archwires and appliances. Given that orthodontic treatment is irreversible, it is difficult to conduct clinical reproducibility trials on the same individual. Due to patient variability and ethical limitations in medical research, there is a lack of objective and effective clinical evidence for the accuracy of customized bracket design and digital IDB. Therefore, it is essential to explore a way to realize it.

In this study, we applied 3D-printed personalized typodonts to simulate the clinical case and employed digital IDB to position customized orthodontic brackets. The objective was to evaluate the precision of digital IDB and personalized orthodontic systems from the perspective of orthodontic outcomes, thereby providing clinical guidance for the promotion of this technology.

## Materials and methods

### Volunteer’s digital information collection

**Fig. 1:**
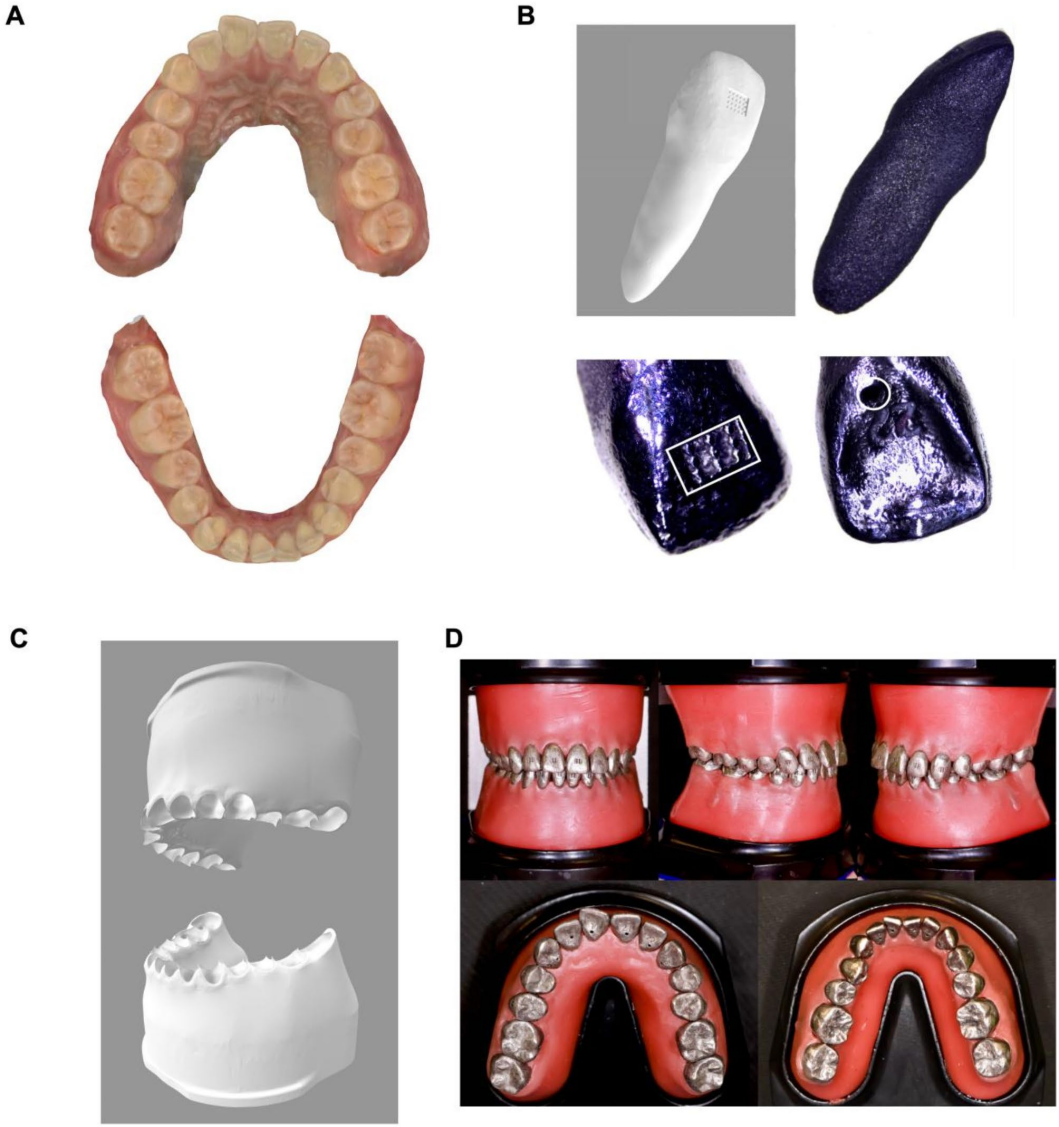
Personalized typodont customization (**A**). Pretreatment intraoral scanning models (T0); (**B**). The personalized tooth was 3D-printed and polished. The undercut structure (the white rectangle) on the labial surface simulated the tooth surface after acid etching, which facilitated stable bonding and positioning of the brackets. The lingual surface featured a circular hole (the white circle) that could overflow internal water-soluble supporting material; (**C**). The digital models of the alveolar bone and alveolar sockets; (**D**). Assembled personalized typodont

One volunteer was chosen for this study and was required to have complete permanent dentition (except third molars), no intraoral implants, no prior orthodontic treatments, and no contraindications to orthodontic treatment, such as progressive temporomandibular disorders or periodontal diseases. The soft and hard tissue data from intraoral and extraoral regions, along with X-ray images, were collected. A 3D digital intraoral scan model was recorded using an intraoral scanner (D2000; 3Shape, Copenhagen, Denmark) as the pretreatment clinical dentition “T0” (Fig. 1, A).

### Personalized typodont customization and accuracy verification

Geomagic Studio software (3D System, Morrisville, NC, USA) was used to segment crown data from “T0”. Mimics software (version 20.0, Materialise, Leuven, Belgium) was employed to extract rough root data from the CBCT through a masking and threshold segmentation process, and then performed surface reconstruction and model optimization. The root and crown data were combined to create personalized 3D tooth models. Individual metal teeth were manufactured with 3D-printing (Carmel 1400c, XJet, Israel) based on these optimized models. The undercut structure on the labial surface simulated the tooth surface after acid etching, which facilitated stable bonding and positioning of the brackets. The lingual surface featured a circular hole that could overflow internal water-soluble supporting material, creating hollow, lightweight teeth without compromising their mobility from their excessive weight (Fig. 1, B). Through Boolean calculation, digital models of the alveolar bone and alveolar sockets were generated (Fig. 1, C). Using 3D-printing (Proxima 6.0, Flashforge, Zhejiang, China), negative resin models were created, which were subsequently converted into wax and used to produce the alveolar bone. Ultimately, the personalized typodont was assembled by inserting the teeth into the waxed alveolar bone (Fig. 1, D). The occlusal contact region of the maxillary dental arch was chosen, the curve surface was thickened by 0.2 mm along the direction of the occlusal surface to form a virtual occluding paper. The occlusal contact area of T0 was obtained by Boolean calculation.

The personalized typodont was scanned, and the digital dental data was recorded as the pretreatment model dentition “T1”. Using Geomagic Control X software (3D Systems, Rock Hill, SC, USA), the “iterative closet point” algorithm was used to achieve the best fit of registration surfaces between T0 and T1. The three-dimensional distance detection was implemented, and the color-scale deviation analysis diagram was generated.

### Design of customized brackets and digital IDB

**Fig. 2:**
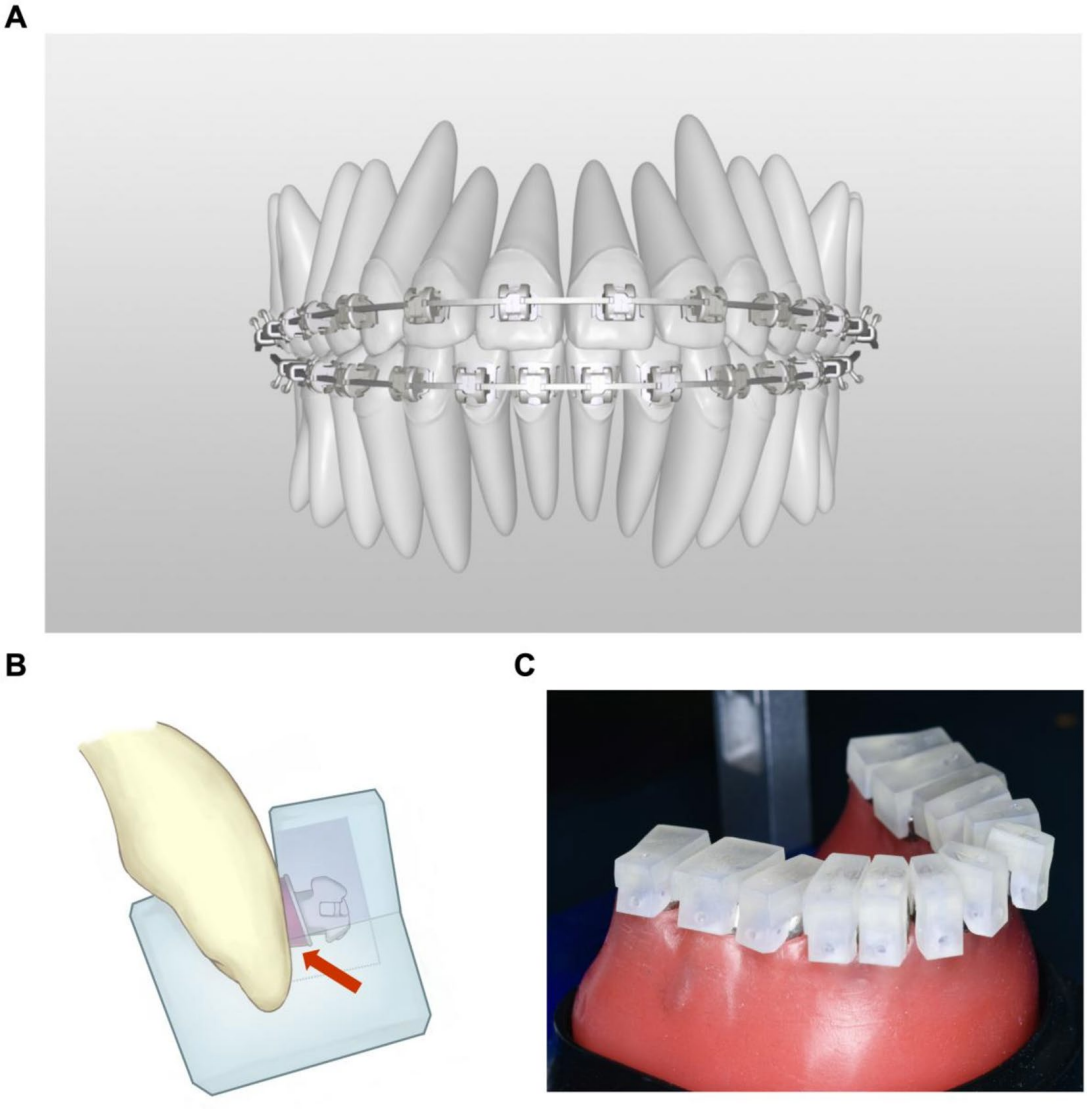
Digital IDB (**A**). Designed the target position (T2) and positioned the virtual brackets; (**B**). The IDB trays were 3D-printed based on the virtual bracket positions and the morphology of crowns. The arrow indicates the individual data-containing metal bracket baseplate; (**C**). Transferred the customized orthodontic brackets to the personalized typodont with IDB trays

The orthodontic target position was determined by two experienced orthodontists jointly, which was named “T2”. Based on T2, the FORMULA software (Shanghai Jing-gong Dental Technology Co., Ltd., Shanghai, China) was used to position a virtual personalized full-sized archwire, and then adjusted the slots of the standardized metal brackets (Damon; Ormco Co., USA) to precisely fit onto the archwire (Fig. 2, A). The space between the base of standardized brackets and the corresponding teeth surface was virtually filled and 3D-printed (WaxJet 400, Flashforge, Zhejiang, China) as the individual data-containing metal bracket bases, which were precisely welded to the standardized brackets to create customized metal brackets. The digital IDB trays which can fully encase the brackets and part of the tooth crowns were 3D-printed (AccuFab-D1s, Shining3D, Zhejiang, China) based on the virtual positions of brackets and the morphology of crowns (Fig. 2, B). The inner layer of the trays was made of silicone rubber, while the outer layer was made of rigid plastic. All archwires used in this experiment were bent by a robot.

### Customized orthodontic process simulation and accuracy verification of outcomes

**Fig. 3:**
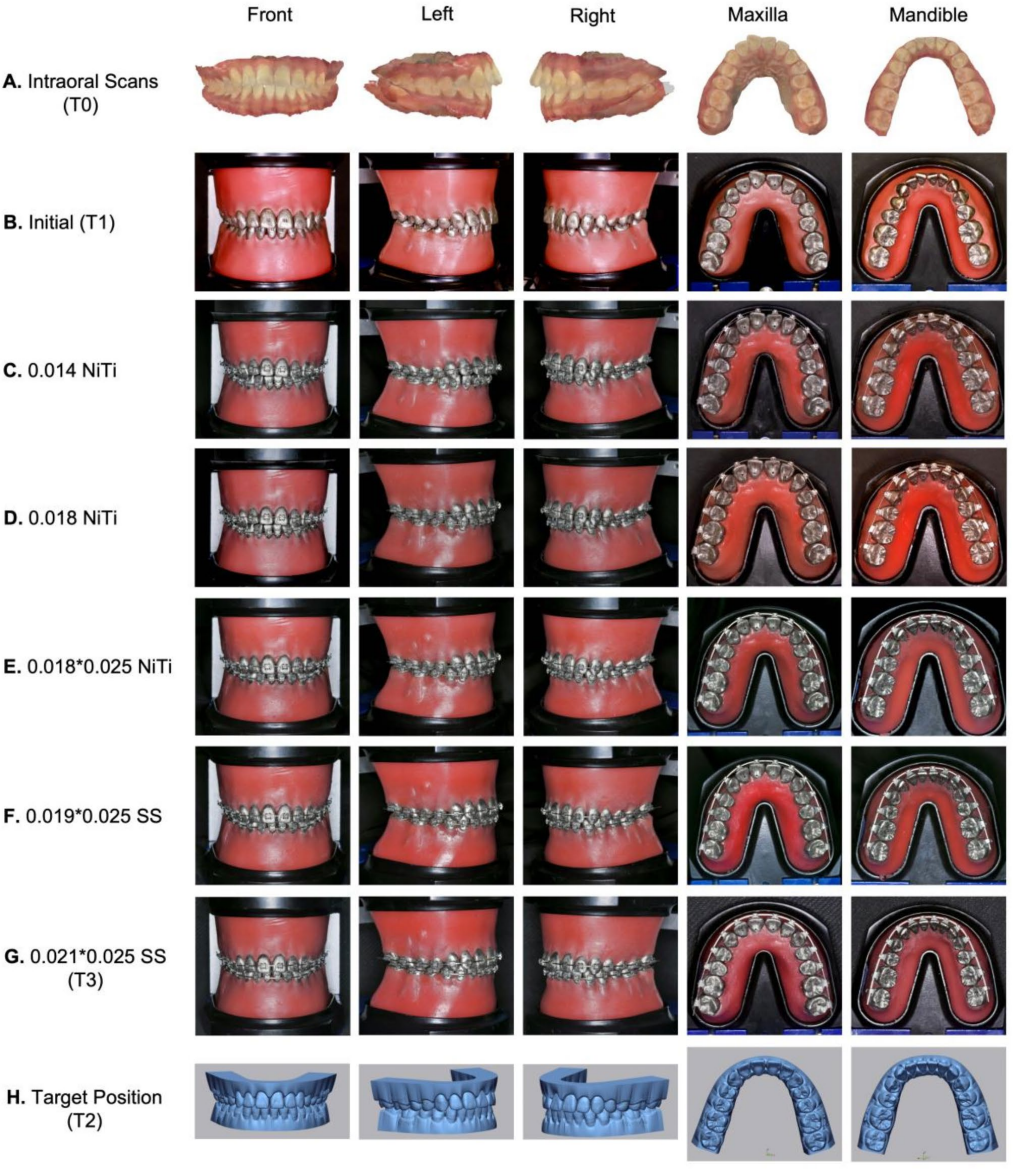
Orthodontic process simulation with personalized typodont (**A**). Pretreatment clinical dentition (T0); (**B**). Pretreatment personalized typodont (T1); (**C**-**G**). Changed the sequential archwires on the typodont to simulate the orthodontic process; (**G**). Actual posttreatment dentition (T3); (**H**). Target position (T2)

The customized orthodontic brackets were bonded to the personalized typodont using digital IDB trays with self-adhesive cement (3M RelyX™ U200 Automix, Minnesota Mining and Manufacturing, China) (Fig. 2, C). The entire orthodontic treatment was carried out using conventional Straight Archwire Techniques. The chosen sequence of customized archwires was as follows: 0.014-in nickel-titanium (NiTi), 0.018-in NiTi, 0.018*0.025-in NiTi, 0.019*0.025-in stainless steel (SS). Considering the potential deviations that may occur with typodonts, we added a 0.021*0.025-in SS archwire, while the bracket slots had dimensions of 0.022*0.028-in [15]. This selection aimed to accurately and fully express the personalized data of the brackets with a full-size archwire. Each archwire was engaged to the baseline setting and heated in a water bath (50℃) for 15 minutes, then cooled in a water bath (0℃) for 5 min. The above steps were repeated until the archwires were fully leveled (Fig. 3). Following the orthodontic simulation, the typodont was scanned, and the digital dental data was recorded as “T3”. The best fit of registration surfaces between T2 and T3 was conducted. Three-dimensional distance detection was then implemented, and the color-scale deviation analysis diagram was generated. The occlusal contact area of T3 was measured using the method described in Sect. “Personalized typodont customization and accuracy verification”.

### Statistical analysis

This study tested and verified the accuracy and repeatability of IDB indirectly via five repetitive experiments with five identical typodonts. Data were analyzed with statistical software (IBM SPSS Statistics, version 27.0; IBM, Armonk, NY, USA). The American Board of Orthodontics Objective Grading System (ABO-OGS) Index, the Orthodontic Peer Assessment Rating (PAR) Index, and the occlusal contact area of T0 and T3 were calculated, and the matching degree of T0-T1 and T2-T3 were evaluated. The whole data conformed to the normality of the distribution and equality of variance. Statistical analysis was performed by the t-test. Statistical significance was assigned at *P* < 0.05 and was 2-tailed.

## Results

### Accuracy verification of personalized typodonts

**Fig. 4:**
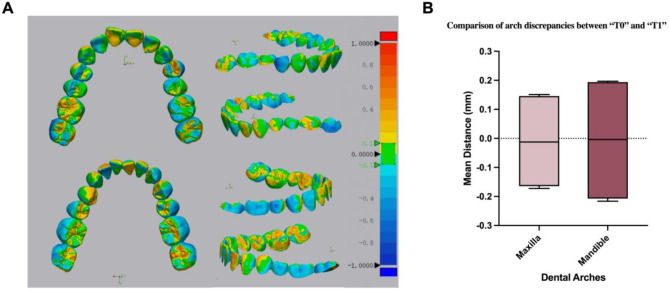
T0-T1 dental arch discrepancies (**A**). Color-scale deviation diagrams; (**B**). Statistical analysis of dental arch discrepancies between T0 and T1

The best-fit surface-based registrations between T0 and T1 were achieved to analyze dental arch discrepancies and generate the color-scale deviation diagrams (Fig. 4). The statistical results were shown in Table 1. The mean discrepancy between the dental arches of the five typodonts and the dentition of patient was 0.15 mm ± 0.01 mm in the maxilla and 0.20 mm ± 0.01 mm in the mandible. Both the maxillary and mandibular dispersion coefficients were within the stability range of 0.6, indicating that the variation between the two dental arches was relatively steady.

**Table 1:** Comparison of dental arch discrepancies between T0 and T1

	+ Mean (mean ± SD) (mm)	- Mean (mean ± SD) (mm)	Maximum (mean ± SD) (mm)	Minimum (mean ± SD) (mm)	RMS	Variation
Maxilla	0.14 ± 0.01	-0.16 ± 0.01	0.51 ± 0.04	-0.51 ± 0.04	0.19 ± 0.01	0.04 ± 0.01
Mandible	0.19 ± 0.00	-0.21 ± 0.01	0.64 ± 0.02	-0.64 ± 0.03	0.25 ± 0.01	0.06 ± 0.01

Note. SD, standard deviation. RMS, root mean square

+ Mean / -Mean, the positive / the negative average deviation between corresponding points of T1 compared to T0

Maximum / Minimum, the maximum deviation between corresponding points of T1 compared to T0

### Orthodontic results evaluation and accuracy verification

After orthodontic treatments with the sequential archwires, the typodonts’ dentition was well-aligned, with normal overbite and overjet, neutral molar relationships, and tight, balanced occlusal contacts. In position T0, the PAR Index was 29 points. In position T3, the average PAR Index of the five groups was 1 point, and the average ABO-OGS Index was 13.8 ± 0.84 points.

**Fig. 5:**
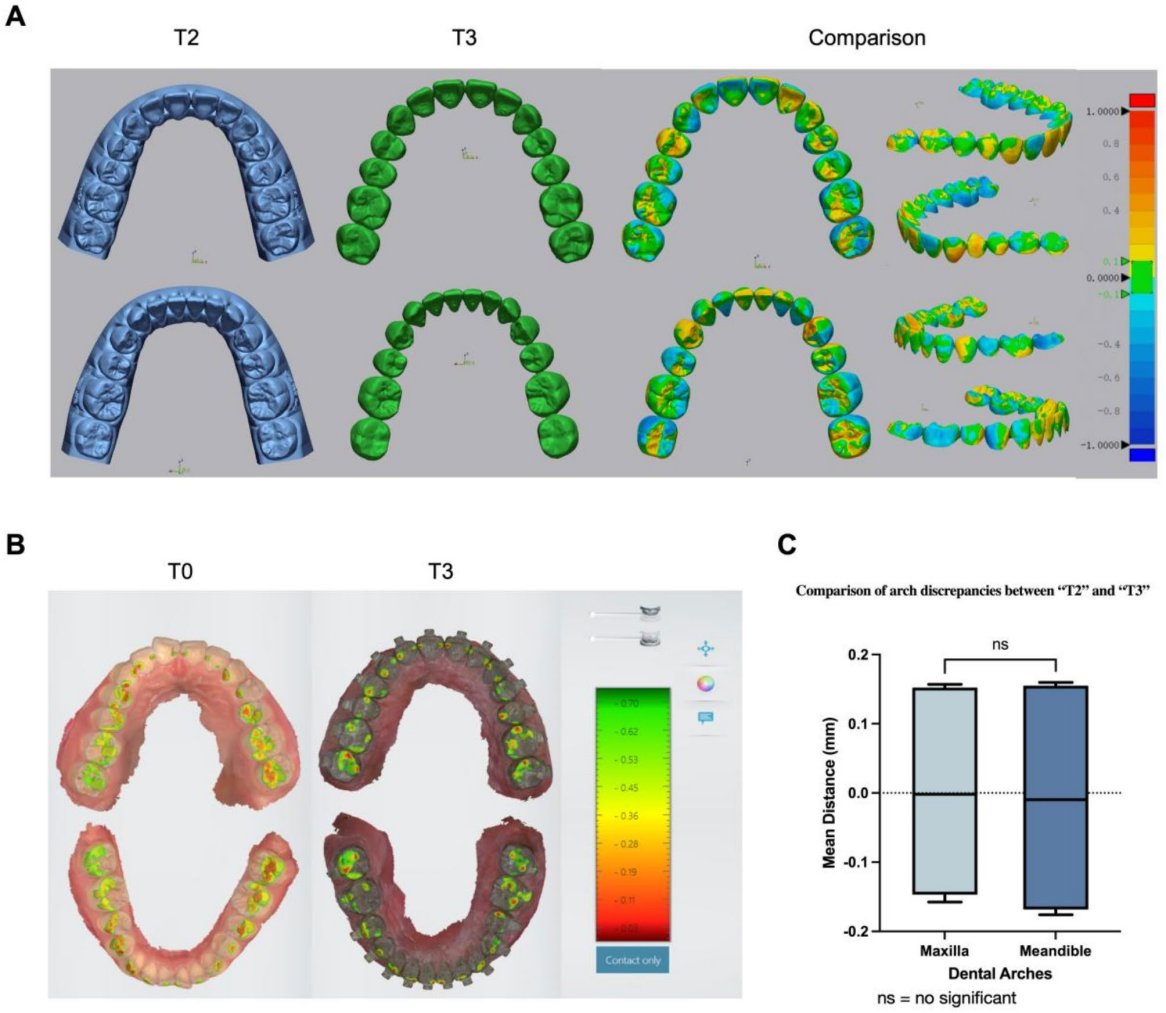
T2-T3 dental arch discrepancies (**A**). The color-scale deviation diagrams; (**B**). The comparison diagrams of the occlusal contact area between T0 and T3. The red areas on the occlusal surface were the actual occlusal contact range within 200 μm in ICP; (**C**). The statistical analysis of dental arch discrepancies between T2 and T3. There was no statistically significant difference between the maxillary and mandibular dentition

The best-fit surface-based registrations between T2 and T3 were achieved to analyze dental arch discrepancies and generate the color-scale deviation diagrams (Fig. 5, A). The statistical results were shown in Table 2. In the maxilla, the mean distance between T2 and T3 was 0.15 mm ± 0.01 mm, the mean root mean square (RMS) was 0.19 mm ± 0.01 mm, while in the mandible, the mean distance was 0.16 mm ± 0.01 mm, the RMS was 0.20 mm ± 0.01 mm.

The occlusal contact area of T0 and T3 in the intercuspal position (ICP) were shown in Fig. 5, B, and the statistical results were shown in Table 3. Before the orthodontic treatment, the occlusal contact area of the left side was significantly larger than the right side. After the orthodontic treatments, the total occlusal contact area increased from 25.24mm^2^ to 29.28mm^2^ ± 1.14mm^2^, with an average change of 4.04mm^2^ ± 1.14mm^2^, and the bilateral occlusal contact area became equally distributed.

**Table 2:** Comparison of dental arch discrepancies between T2 and T3

	+ Mean (mean ± SD) (mm)	- Mean (mean ± SD) (mm)	Maximum (mean ± SD) (mm)	Minimum (mean ± SD) (mm)	RMS	Variation
Maxilla	0.15 ± 0.01	-0.15 ± 0.01	0.74 ± 0.06	-0.73 ± 0.02	0.19 ± 0.01	0.04 ± 0.01
Mandible	0.15 ± 0.01	-0.16 ± 0.01	0.64 ± 0.03	-0.77 ± 0.04	0.20 ± 0.01	0.04 ± 0.00
*P* Value	0.909					

Note. *P* Value is the deviation of mean variation between maxilla and mandible

SD, standard deviation. RMS, root mean square

+ Mean / -Mean, the positive / the negative average deviation between corresponding points of T3 compared to T2

Maximum / Minimum, the maximum deviation between corresponding points of T3 compared to T2

**Table 3:** The occlusal contact area of pretreatment and posttreatment

	Anterior Region (mean ± SD) (mm2)	Left Postierior Region (mean ± SD) (mm2)	Right Postierior Region (mean ± SD) (mm2)	Total Region (mean ± SD) (mm2)
Before Treatment	5.65	13.29	6.3	25.24
After Treatment	7.78 ± 0.88	10.28 ± 0.81	11.23 ± 0.38	29.28 ± 1.14

Note. SD, standard deviation

## Discussion

In this study, a 3D-printed personalized typodont system was developed, tested and successfully implemented. For customized orthodontic systems that utilize straight-wire technology, the accuracy of brackets’ placement was critically important to the orthodontic outcome. The matching degree between the target position and the actual final position in customized orthodontic systems reflected the accuracy of bracket bonding, thereby demonstrating the accuracy and reproducibility of IDB. This study is the first to indirectly evaluate the accuracy and repeatability of digital IDB from the perspective of orthodontic outcomes.

Traditional typodonts provide additional technical assistance for the research of orthodontic biomechanics and the prediction of the accuracy of orthodontic effectiveness. The teeth on the typodonts can move swiftly when the wax is softened in the warm water bath, allowing them to simulate the orthodontic procedures, forecast and assess the final therapeutic outcomes [16, 17]. However, several limitations cannot be ignored. First, heat conduction in the deep wax arch is poor. Second, traditional typodonts with standard teeth and wax arches can hardly reflect real clinical conditions. Third, various biological and biomechanical factors may influence the actual results. Researchers have made efforts to address these issues, such as using special materials as the base, ensuring uniform wax thickness, and employing the Heat Induction Typodont System (HITS) [18–20]. Nonetheless, suitable materials are difficult to find, and the wires connected to the HITS may interfere with the tooth movements [21]. Techniques such as the FEM and the Orthodontic Measurement and Simulation System have been used to forecast tooth movement through mechanical analysis. However, due to aeolotropism, non-linear elasticity and individual patient differences, it is challenging to perform accurate dynamic modeling [18]. As a result, it is impossible to truly reproduce clinical results in different patients.

In this study, we developed a novel typodont model with personalized metal teeth and alveolar bone by 3D-printing. The metal material facilitated heat conduction, allowing the roots wrapped around in wax to be easily moved, thereby better simulating the effect of orthodontic forces. The best-fit surface-based registrations between T0 and T1 were achieved. The RMS was computed to assess the 3D deviation between the 3D positions of two aligned models. Kuang BY [22] and Han XR [23] contended that if the RMS value of the mean distance between two models is less than 0.3 mm, there is high registration accuracy and the error can be disregarded. Both the mandibular and maxillary RMS in our investigation fell inside this range, which indicated that the typodonts had excellent imitation degrees. The typodonts we produced could simulate natural dentitions and the relative positions of the alveolar bone and tooth roots. This approach makes it possible to repeat orthodontic treatments and achieve orthodontic visualization within the same clinical case. Simulating orthodontic treatments with personalized typodonts makes it possible to evaluate the process and predict the outcomes accurately. This allows orthodontists to modify treatment schemes timely, reduce orthodontic risks, and ultimately result in optimal outcomes. Apart from unchangeable factors such as patient compliance and the density of alveolar bone, the position of brackets and the resistance of teeth movement in wax also lead to deviations between simulation and outcomes. Using full-sized archwires can minimize the impact of wax resistance on tooth movement, thereby reducing the differences between typodonts and actual clinical scenarios. In this study, we used hollow, lightweight metal teeth, personalized typodonts and full-sized archwires to minimize the discrepancies between simulation and actual outcomes.

The accuracy of direct bonding (DB) is limited by the subjective opinions, technological proficiency and clinical experience of the orthodontist, which can easily result in improper bracket positioning and reduce orthodontic efficiency. Some researchers supported that IDB is more predictable and precise than DB, with shorter clinical chairside time, although the overall procedure is longer and more expensive [5, 24, 25]. Traditional IDB determines bracket positions using the gypsum casts [26]. With the establishment of three-dimensional reconstruction technology, digital IDB has overcome many limitations of gypsum models, including its time-consuming manufacturing process, challenging preservation techniques, and inaccurate measurement results [27]. This allows orthodontists to easily modify orthodontic schemes and bracket positions. Previous studies have reported that digital IDB trays are more accurate than traditional IDB trays, offering additional advantages in terms of positioning brackets in three dimensions and angles [26, 28]. On the contrary, others have claimed that the accuracy of digital trays is lower than that of conventional trays, although both trays show good results [29]. Several decades ago, the digital technique was considered less precise in mapping the tips of dental cusps, a critical reference point for determining bracket position [30]. With the continuous development of digital technology, the errors in current intraoral scanning technology have decreased [31]. Meanwhile, increasing the contact area between the trays and the teeth allows the trays to be placed more stably, enhancing the accuracy of bracket bonding.

In this study, digital IDB was used to position customized brackets, and personalized typodonts were placed in a water bath to simulate straight-wire orthodontic procedures. The accuracy of digital IDB was verified through repetitive experiments. Considering that the mean distance between two dental arches can more effectively describe differences than linear measurements [8], this study used the holistic matching degree to assess discrepancies between the posttreatment typodont and the presumptive target position. Armstrong et al. [32] proposed that deviations in bracket positions for maxillary central incisors and mandibular incisors by more than 0.25 mm or for other teeth by more than 0.5 mm, can result in different clinical orthodontic outcomes. Additionally, the ABO-OGS index specifies that 0.5 mm is the standard for both tooth alignment and marginal ridge irregularity [33]. In this study, all deviations in the parallel experiments fell below this upper limit. The ABO-OGS index, the PAR index, and the occlusal contact area from the parallel experiments all demonstrated the accuracy, reliability, and high repeatability of the results from the customized orthodontic system. The criteria of the ABO-OGS index are more detailed and stringent than the PAR index. According to the ABO-OGS index, some conditions such as irregular tooth alignment and irregular marginal ridge require repeated scoring for the adjacent teeth, which can lead to a higher score. In this study, the mean ABO-OGS index score following treatment was 13.8 ± 0.84, fulfilling the criteria for satisfactory cases (within 15 points). These results demonstrate the accuracy of the simulated orthodontic outcomes, indirectly confirming the accuracy of digital IDB and the repeatability of the bracket bonding positions. Previous studies [34–36] have mostly evaluated digital IDB by contrasting three-dimensional directional discrepancies between intended and actual bracket positions. Recently, an in vitro study [37] showed that, with IDB, the mean distance error of the bracket bonding locations was 0.09 mm, and the mean height error was 0.15 mm. Another in vivo study [38] using IDB found that neither the mean nor single linear deviations exceeded the set cutoff value of 0.25 mm. This research is the first to demonstrate the accuracy of digital IDB from the perspective of orthodontic outcomes, and the results tied well with previous studies.

One limitation of this research is that this sample was a non-extraction patient with relatively low orthodontic difficulties, minimal tooth movements toward the target position, and a high degree of treatment success. In subsequent phases, our team will increase the complexity of the orthodontic cases, including those involving tooth extraction and substantial root movement, to further validate the accuracy of 3D tooth movement simulation with this technology. This will further verify the accuracy of digital IDB and the customized orthodontic system.

## Conclusion

In this study, we used personalized typodonts to simulate orthodontic procedures, which had the advantage of visualization and repeatability in verifying orthodontic efficacy. The results indicated that using digital IDB to bond customized orthodontic brackets on personalized typodonts can accurately achieve the desired orthodontic outcomes, with the final results closely matching the expected target positions. Overall, this research is the laboratory support for future explorations of IDB and customized orthodontic systems. As goal-guided, individualized therapeutic techniques, they offer the advantages of visualization and predictability in treatment processes. These techniques have a broad scope in future clinical applications, providing a more precise and reliable method for orthodontic treatment.

## Data Availability

The datasets used and analysed during the current study are available from the corresponding author on reasonable request.
